# The 36-Month Survival Analysis of Conservative Treatment Using Platelet-Rich Plasma Enhanced With Injectable Platelet-Rich Fibrin in Patients With Knee Osteoarthritis

**DOI:** 10.7759/cureus.35632

**Published:** 2023-03-01

**Authors:** Vorasilp Cheeva-akrapan, Thana Turajane

**Affiliations:** 1 Biotechnological Research and Development Center, Police General Hospital, Bangkok, THA

**Keywords:** orthobiologics, survival rate, knee osteoarthritis, platelet-rich fibrin, platelet-rich plasma

## Abstract

Introduction: Knee osteoarthritis (KOA) is a musculoskeletal disease that leads to pain, stiffness, and deformity of the load-bearing knee joints. Biologic products including platelet-rich plasma (PRP) and platelet-rich fibrin (PRF) are now in the spotlight for the treatment of KOA owing to their role of disease-modifying potential effect. There are still limited studies on the survival rate of KOA treated with biological intervention. We conducted this study to evaluate the primary outcome as the survival rate of KOA treated with PRP enhanced with injectable PRF that helps avoid unnecessary surgical intervention.

Methods: There were 368 participants who met the inclusion and exclusion criteria. Participants were informed about this prospective cohort study protocol and signed written consent forms. Each participant received one injection of 4 ml of PRP and 4 ml of injectable PRF (iPRF), the so-called “PRP enhanced with iPRF”. Clinical assessment was evaluated with the visual analog scale (VAS) at the second, fourth, sixth, 12th, 18th, 24th, 30th, and 36th-month post-treatment. If the VAS pain score improved by more than 80% compared to the previous treatment, there was no need for a repeated dose. If the pain scores improved by 50% to 80% compared to the previous treatment, the participants were advised a repeated dose. However, if the pain scores improved by less than 50% compared to the previous treatment, the participants were advised to undergo surgical intervention instead of a repeated dose. The primary outcome was recorded as any surgical treatment (either arthroscopic knee surgery, unicondylar arthroplasty, or total knee arthroplasty) at any time point post-treatment. The secondary outcome was recorded as the interval (months) between first-to-second, second-to-third, and third-to-fourth injections.

Results: The overall survival rate of knees that did not require surgical intervention during the 36-month follow-up was 80.18%. The mean number of injections was 2.52±0.07 times for overall participants. The mean interval time was 5.42±0.36, 8.92±0.47, and 9.58±0.55 months for the first-to-second, second-to-third, and third-to-fourth injections.

Conclusion: This study supports the usage of PRP enhanced with iPRF as a biologic modality for the treatment of KOA. This treatment modality provides a satisfactory survival rate at the 36-month follow-up. The longer interval between each injection supports the disease-modifying effect of PRP enhanced with iPRF.

## Introduction

Knee osteoarthritis (KOA) is a musculoskeletal disease that leads to pain, stiffness, and deformity of the load-bearing knee joints [1]. The prevalence of KOA is now surging due to the longer longevity of people, the more desire for sport recreation, and a healthier lifestyle. This causes osteoarthritis as one of the diseases that contribute to higher years lived with disability [2]. The pathogenesis of KOA has long been believed as mechanic-in-origin. Latest studies have shown that cellular and molecular factors also play a major role in disease progression which include synovial inflammation, and pathological chondrocyte catabolic responses leading to cartilage degradation [3]. Up until today, the treatment for KOA ranges from patient education, physical rehabilitation, oral medication, intra-articular corticosteroid or hyaluronic acid injection, and surgery as the last resort [4-5]. Oral medication may lead to gastrointestinal, renal, or cardiovascular side effects [6]. Due to the cellular toxicity effect of corticosteroid usage, long-term usage of this modality should be avoided [7]. Burnett et al. showed that there is a dose-dependent effect of intra-articular corticosteroid injection on the risk for total knee arthroplasty (TKA) at the five-year follow-up [8]. The injection of hyaluronic acid provided a 71.6% survival rate at the three-year follow-up [9]. Meanwhile, surgery such as TKA provided an 82.3% survival rate for a 25-year survival study [10]. The aforementioned treatment modalities mostly focus on symptomatic pain relief instead of the cause of pain generator. This brings the use of biological products into the spotlight for the treatment of KOA owing to their disease-modifying potential.

Platelet-rich plasma (PRP), an autologous blood condition that is processed through the spinning method, has been proposed as a new paradigm for the treatment of KOA [11]. The PRP exerts its therapeutic mechanism via growth factors stored in the α-granule of platelets [12-13]. Platelet-rich plasma has been proven to decrease inflammation, produce high-quality synovial fluid, and reconstruction of degenerative cartilage [14]. However, with the addition of an exogenous activator, PRP is thought to release the growth factors so quickly that there is not enough quality left for the healing process when cells emerge from the surrounding tissue [15]. 

Platelet-rich fibrin (PRF), another kind of autologous blood condition prepared without the use of an anticoagulant, was initially invented to be used as a scaffold in the maxillofacial field [16]. The slow fibrin polymerization during the PRF spinning method provides the trapping of platelet and cytokines within its meshwork. This leads to the progressive release of the functioning molecules during the fibrin matrix remodeling process [17]. Here, we process patients’ own blood as injectable PRF (iPRF) to be used in conjunction with PRP to prolong the treatment effect.

There are still limited studies on the survival rate of KOA treated with biological intervention. We conducted this study to evaluate the primary outcome as the survival rate of KOA treated with PRP enhanced with iPRF to avoid unnecessary surgical intervention. The secondary outcome in this study was the number of injections and the interval time between each injection.

## Materials and methods

A prospective cohort study was performed at the Biomedical Technology Research and Development Centre, Police General Hospital, Bangkok, Thailand from February 2018 to June 2022 after approval from the internal review board and hospital ethics committee (approval no. 49/2019). The sample size was calculated based on a related study with an alpha of 0.05 and a power of 80% [18]. The calculated sample size was 524 knees divided into 424 knees in Kellgren and Lawrence (KL) grade I to III, and 100 knees in KL grade IV groups.

Patients with complaints of knee pain at the outpatient orthopedic clinic were evaluated for eligibility in the current study. The inclusion criteria were: patients aged more than 65 years diagnosed with KOA classified as KL grade I to IV, those who failed conservative treatments including no response to oral medications for at least six months, physiotherapy for at least three sessions, intra-articular injection of steroid or hyaluronic acid (at least one dose), hemoglobin (Hb) levels >11 g/dL, and platelet count >80,000 cells/µL. The exclusion criteria included patients diagnosed with meniscal or knee ligament injury, inflammatory arthritis, systemic bleeding disorder, those who presented with radiographic deformity of tibiofemoral angle more than 5 degrees, and those with a history of nonsteroidal anti-inflammatory drugs (NSAIDs) usage within five days of blood drawn or intake of anticoagulant or anti-aggregate drugs.

A total number of 368 participants met the criteria. The participants were informed about the study protocol and signed written consent forms.

PRP & PRF preparation

A 30 ml peripheral blood sample was collected from each participant. The first 20 ml of blood was separated in two centrifuge tubes (PP&GF, Bangkok, Thailand) with 10 ml for each tube to mix with acid citrate dextrose anticoagulant. The blood was centrifuged using the ALPAS centrifugation machine (Bangkok, Thailand) at 250 G for six minutes. After the first spin, the blood was separated into three components: red blood cells on the bottom layer, a buffy coat in the middle layer, and platelet-containing plasma at the top layer. The upper two layers were gently aspirated and transferred to a new tube and centrifuged again at 1000 G for 10 minutes. After the second spin, the platelet-poor plasma at the upper layer was gently aspirated for removal. Around 1 ml of the residual, leukocyte-rich PRP was collected for a complete blood count. The remaining 4 ml of PRP was aspirated using a 5 ml sterile injection syringe for intra-articular knee injection. The remaining 10 ml of peripheral blood was transferred to the last tube and was centrifuged once with 250 G for six minutes to produce 4 ml of iPRF.

Injection protocol

A well-trained team of orthopedic attendings and residents performed all intra-articular knee injections. The participant was in a supine position with the knee flexed at 90 degrees. The site of injection was the anteromedial joint space. An antiseptic agent was applied to the skin. The analgesic agent was infiltrated into the skin and subcutaneous tissues surrounding the injection site with a 25G needle. Air-test was performed with an 18G needle to confirm the position of the needle in the intra-articular space. Both PRP and iPRF were delivered to the joint space with the same needle. The knee was immediately extended after the biological agent was delivered. The participant was allowed to perform full weight bearing after injection.

Rehabilitation protocol

The participant was advised to perform fixed arc quadriceps exercise two days after the injection. The participant sat on a chair with their legs extended forward for 100 seconds on each side. This exercise was recommended to be performed three to five times daily.

Follow-up assessment

Participants were assessed at the second, fourth, sixth, 12th, 18th, 24th, 30th, and 36th-month post-treatment. If the VAS pain score improved by more than 80% compared to the previous treatment, there was no need for a repeated dose. If the pain scores improved by 50% to 80% compared to the previous treatment, the participants were advised to repeat the dose. However, if the pain score improved by less than 50% or the pain experienced deteriorated compared to the pre-treatment condition, the participants were advised surgical intervention instead of a repeated dose. Participants were advised of common post-injection reactions that might occur which included tightness in the knee and swelling within two weeks post-injection. 

Statistical analysis

The primary outcome was recorded as any surgical treatment (either arthroscopic knee surgery, unicondylar arthroplasty, or TKA) at any time point post-treatment. The secondary outcome was recorded as the interval (months) between first-to-second, second-to-third, and third-to-fourth injections. Continuous data were elaborated in mean and standard deviation (SD), and discrete data were elaborated in percentage and proportion. Survival analysis with monthly interval units was used to determine the event rate. The time at risk was the number of months that the participants were followed up with an endpoint of 36 months. Right truncation, left truncation, right censoring, and left censoring protocols were used. The Kaplan-Meier curve was used to illustrate the primary outcome as the percentage of survival at each time point. The difference in survival function was determined by the log-rank test for equality of survivor function. A p-value of <0.05 was considered statistically significant. Stata software (StataCorp LLC, College Station, TX, USA) version 16.0 was used for statistical analysis.

## Results

A total number of 378 participants were included. Ten participants were lost to follow-up, thus leaving 368 participants completing the current study. The demographic data of participants are demonstrated in Table 1.

**Table 1:** Demographic characteristics of participants in the study N: Number

	KL I-III (group 1)	KL IV (group 2)	Total
N (Knees)	321	47	368
Age (Years) (Mean±SD)	69.34±0.48	70.19±1.06	69.45±0.44
Gender (N, Female:Male)	261:60	35:12	296:72
Site (N, Left:Right)	147:174	23:24	170:198

**Figure 1:**
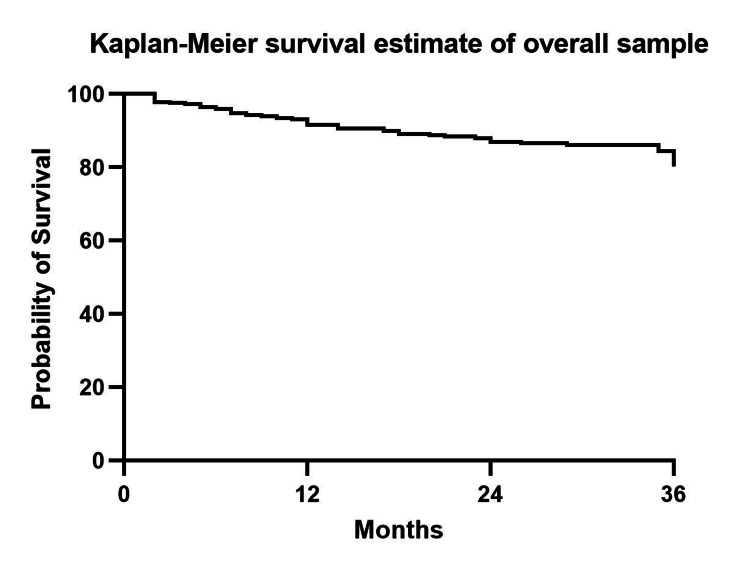
The Kaplan-Meier curve shows the survival estimate rate of KOA participants not undergoing any surgical intervention after treatment of PRP enhanced with iPRF intra-articular injection. KOA: Knee osteoarthritis, PRP: Platelet-rich plasma, iPRF: Injectable platelet-rich fibrin

**Figure 2:**
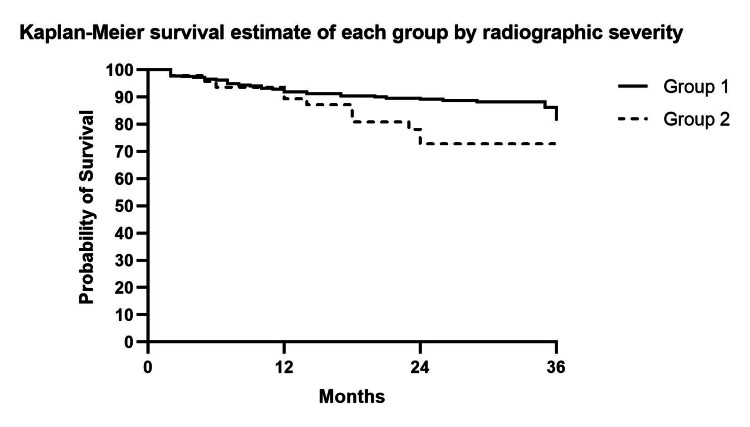
The Kaplan-Meier curve shows the survival estimate rate of KOA participants not undergoing any surgical intervention after treatment of PRP enhanced with iPRF intra-articular injection categorized by radiographic severity. The thick line represents participants with radiographic KL I-III (group 1). The dotted line represents participants with radiographic KL IV (group 2). KOA: Knee osteoarthritis, PRP: Platelet-rich plasma, iPRF: Injectable platelet-rich fibrin, KL: Kellgren and Lawrence

The overall survival rate of knees that did not require surgical intervention during the 36-month follow-up was 80.18% (Figure 1). We performed a subgroup analysis based on the severity of the radiographic findings of knees classified with the KL classification. The first group included participants with radiographic KL grade I to III (group 1) while group 2 included those with KL grade IV classification. The log-rank test was used to analyze the difference in survival rates between the two groups. The survival rates at the 36th-month follow-up (Figure 2) were 81.38% and 72.77% for group 1 and group 2, respectively (p-value=0.03).

The mean number of injections was 2.52±0.07 times for overall participants. The mean number of injections for group 1 and group 2 were 2.47±0.73 and 2.87±0.22, respectively (p-value=0.05). Regarding the interval between each injection, the mean interval time was 5.42±0.36, 8.92±0.47, and 9.58±0.55 months for the first-to-second, second-to-third, and third-to-fourth injections.

## Discussion

The interesting finding in this study was that PRP enhanced with iPRF could save the osteoarthritic knee from surgical intervention up to 80.18% by the 36th-month follow-up. The longer interval time for each consecutive injection was supported by the theory of iPRF functioning as a natural scaffold [17]. The combination of PRP and iPRF enhances the release of active molecules at each time point. The PRP releases growth factors immediately after injection while iPRF functions as a natural meshwork for PRP and progressively releases growth factors. Shaoheng et al. in 2019 performed a study of fat grafting using PRP and PRF. It showed that PRF provided better tissue retention, quality, and vascularization when compared to PRP [19].

Another interesting point in this study is the inclusion of patients with radiographic osteoarthritis KL grade IV who were normally excluded from the study of biological treatment for KOA. The finding demonstrated that patients with KOA KL IV also responded to biological treatment. However, the survival rate was still lower than those in the less severe group. Hunter et al. found that the prevalence of radiographic KOA was higher than the prevalence of symptomatic KOA [20]. We propose that radiographic osteoarthritis is not related to symptomatic osteoarthritis. Thus, patients with severe radiographic osteoarthritis are still eligible for biologic treatment.

For the treatment of KOA, patient education is still mandatory. In this study, the follow-up session was mainly to insist on the importance of home-based rehabilitation protocol. With this continuous education, patients were likely to comply with the home-based program and benefit from a better outcome of treatment with biological agents. Negrini et al. in 2021 published a case report on rehabilitation after intra-articular platelet-rich growth factors injection in patients with KOA. The results showed that the pain and functional abilities were improved at the two-month follow-up [21]. 

The repeated dose for PRP enhanced with iPRF was questionable. In the current study, we performed one injection for each cycle of biological treatment leading to the mean number of 2.5 injections for the whole 36-month treatment interval. The meta-analysis by Vilchez-Cavazos et al. in 2019, demonstrated that single and multiple injections of PRP provided the same pain improvement in patients with KOA [22]. With the health care service after the COVID-19 pandemic, we agreed that patients would like to visit the hospital less. We provided one injection of PRP enhanced with iPRF to prolong the treatment effect of our treatment. 

There were limitations in this study. Firstly, this was a prospective cohort study. The PRP treatment for KOA is not standard treatment in this country, so a randomized controlled trial could not be performed. Secondly, this study focused mainly on patient outcome evaluation. We did not perform pathological improvement. In further studies, we suggest the study of cartilage regeneration. Thirdly, we could not reach the expected number of participants as calculated by sample size. Due to the COVID-19 pandemic, there were a limited number of patients encountering healthcare providers in our country since 2019. With future studies, we expect to recruit more patients as participants.

## Conclusions

The treatment of KOA with biological treatment such as PRP enhanced with iPRF can be considered as a disease-modifying modality which in turn treats the cause of KOA. Platelet-rich plasma enhanced with iPRF provided an 80.18% survival rate for patients with KOA at the 36th-month follow-up. This biologic modality can be used to treat patients with KOA regardless of their radiographic severity. The iPRF should be injected in conjunction with PRP to prolong effective treatment time.
